# The Role of the Multiple Banded Antigen of *Ureaplasma parvum* in Intra-Amniotic Infection: Major Virulence Factor or Decoy?

**DOI:** 10.1371/journal.pone.0029856

**Published:** 2012-01-12

**Authors:** Samantha J. Dando, Ilias Nitsos, Suhas G. Kallapur, John P. Newnham, Graeme R. Polglase, J. Jane Pillow, Alan H. Jobe, Peter Timms, Christine L. Knox

**Affiliations:** 1 Institute of Health and Biomedical Innovation, Queensland University of Technology, Brisbane, Queensland, Australia; 2 School of Women's and Infants' Health, University of Western Australia, Perth, Western Australia, Australia; 3 Department of Neonatology and Pulmonary Biology, Cincinnati Children's Hospital Medical Center, University of Cincinnati, Cincinnati, Ohio, United States of America; Columbia University, United States of America

## Abstract

The multiple banded antigen (MBA) is a predicted virulence factor of *Ureaplasma* species. Antigenic variation of the MBA is a potential mechanism by which ureaplasmas avoid immune recognition and cause chronic infections of the upper genital tract of pregnant women. We tested whether the MBA is involved in the pathogenesis of intra-amniotic infection and chorioamnionitis by injecting virulent or avirulent-derived ureaplasma clones (expressing single MBA variants) into the amniotic fluid of pregnant sheep. At 55 days of gestation pregnant ewes (n = 20) received intra-amniotic injections of virulent-derived or avirulent-derived *U. parvum* serovar 6 strains (2×10^4^ CFU), or 10B medium (n = 5). Amniotic fluid was collected every two weeks post-infection and fetal tissues were collected at the time of surgical delivery of the fetus (140 days of gestation). Whilst chronic colonisation was established in the amniotic fluid of animals infected with avirulent-derived and virulent-derived ureaplasmas, the severity of chorioamnionitis and fetal inflammation was not different between these groups (p>0.05). MBA size variants (32–170 kDa) were generated *in vivo* in amniotic fluid samples from both the avirulent and virulent groups, whereas *in vitro* antibody selection experiments led to the emergence of MBA-negative escape variants in both strains. Anti-ureaplasma IgG antibodies were detected in the maternal serum of animals from the avirulent (40%) and virulent (55%) groups, and these antibodies correlated with increased IL-1β, IL-6 and IL-8 expression in chorioamnion tissue (p<0.05). We demonstrate that ureaplasmas are capable of MBA phase variation *in vitro*; however, ureaplasmas undergo MBA size variation *in vivo*, to potentially prevent eradication by the immune response. Size variation of the MBA did not correlate with the severity of chorioamnionitis. Nonetheless, the correlation between a maternal humoral response and the expression of chorioamnion cytokines is a novel finding. This host response may be important in the pathogenesis of inflammation-mediated adverse pregnancy outcomes.

## Introduction

The two *Ureaplasma* species, which cause infections in humans are *Ureaplasma parvum* (serovars 1, 3, 6 and 14) and *Ureaplasma urealyticum* (serovars 2, 4, 5, 7–13) [1]. Phenotypically the ureaplasmas are distinguished from the closely related *Mycoplasma* species by their ability to hydrolyse urea to generate 95% of their ATP [2], [3]. The ureaplasmas are generally regarded as commensals of the lower genital tract in both males and females and can be isolated from the vagina or cervix in 40–80% of sexually active females [4], [5]. However, ureaplasma infection of the upper genital tract during pregnancy is associated with adverse pregnancy outcomes including preterm birth and chorioamnionitis [5], [6].

Ureaplasmas are hypothesized to gain access to the upper genital tract of pregnant women by various mechanisms including (i) ascending invasive infection from the lower genital tract; (ii) transplacental or haematogenous spread; or (iii) iatrogenic needle contamination at the time of amniocentesis or chorionic villous sampling [7]. Although ureaplasmas are the bacteria most frequently isolated from infected amniotic fluid (AF) in pregnant women [8]–[10], the pathogenic role of these microorganisms during pregnancy is unclear, as ureaplasmas have also been isolated from the AF of women with apparently normal pregnancy outcomes after delivery at term [8], [9], [11]. These discrepancies demonstrate that a causal relationship has not been established between intra-amniotic ureaplasma infection and adverse pregnancy outcomes.

Initial serotyping studies of invasive ureaplasmas isolated from CSF and blood cultures of neonates demonstrated that no one serovar was more associated with disease, and that invasiveness was not likely to be limited to one particular serotype [12]. Rather, it was hypothesised that the virulence of individual ureaplasma strains may be determined by antigenic variation and/or host factors [5], [12]. The multiple banded antigen (MBA) is a surface exposed lipoprotein, which can undergo size and phase variation *in vitro* and *in vivo* [13]–[17]. The MBA gene (*mba*) consists of a 5′ conserved region, which encodes a signal peptide and membrane anchor and a 3′ repetitive region, which consists of multiple tandem repeat units [14]. The MBA is predicted to be a major ureaplasmal virulence factor and is the predominant antigen recognised by sera during infections in humans [18]. Recently, our group demonstrated that MBA size variation was associated with the severity of histological chorioamnionitis in a pregnant sheep model of intra-amniotic ureaplasma infection [17]. From this previous work, we cultured a clonal *U. parvum* serovar 6 virulent-derived strain (associated with severe histological chorioamnionitis) and a clonal avirulent-derived strain (associated with no signs of histological chorioamnionitis), which we aimed to characterize further *in vivo*.

Whilst it is evident that certain ureaplasma isolates are more associated with severe disease than others, it is not known if the invasive properties associated with these isolates are determined primarily by bacterial factors (such as size/phase variation of the MBA) or host factors (including the immune response). Using our established sheep model of chronic intra-amniotic ureaplasma infection, we tested whether clonal ureaplasma isolates with defined MBA profiles (derived from virulent or avirulent parent strains, associated with either severe, or no chorioamnionitis) are intrinsically virulent or avirulent. For this study we defined virulence as the extent of damage to the host during infection with a pathogen, as defined by Brown *et al.* [19]. We hypothesized that virulence is not likely to be associated with specific ureaplasma isolates, but rather that the severity of disease may be determined by the host immune response generated against intra-amniotic ureaplasma infection. We predicted that interactions between ureaplasmas and the host immune response may be mediated by size/phase variation of the MBA and that variable expression of the MBA would enable ureaplasmas to avoid eradication by host immune factors. By measuring pregnancy/fetal outcomes, ureaplasma colonization of fetal tissues, MBA *in vivo* expression profiles and the host immune response we aimed to provide insight into the role of the MBA during microbial invasion of the amniotic cavity and the chorioamnion.

## Materials and Methods

### Ethics Statement

This study was carried out in accordance with the NHMRC ‘Australian code of practice for the care and use of animals for scientific purposes’ and approved by the UWA Animal Ethics Committee (Approval No. RA/3/100/619). Preterm lambs were surgically delivered by Caesarean section. Ewes were pre-medicated with an intra-venous injection of ketamine (10 mg/kg bodyweight) and medetomidine (0.02 mg/kg) and a subdural injection of 2% lignocaine (60 mg). The fetus was delivered and then euthanized using sodium pentobarbitone, 100 mg/kg. The ewe was killed by sodium pentobarbitone, 100 mg/kg.

### Source of ureaplasma isolates

The *U. parvum* serovar 6 strains used for intra-amniotic injection were expanded from single colony forming units (CFUs) derived from ‘virulent’ and ‘avirulent’ ureaplasmas isolated from the AF of pregnant sheep from our previous experiment [17]. The parent strains (E24 and E22) were classified as virulent and avirulent respectively, based on the severity of chorioamnionitis associated with intra-amniotic infection and the number of MBA variants detected within the AF at the time of preterm delivery. Infection with isolate E24 was associated with severe chorioamnionitis (resulting in fibrosis and tissue lesions) and a low number of AF MBA size variants (n = 5); whereas infection with isolate E22 did not result in histological chorioamnionitis and a greater number of MBA size variants were detected within the AF (n = 14, data presented in reference [17]). For this current experiment, to obtain ureaplasmas expressing single MBA size variants derived from single CFUs, E22 and E24 cultures were cloned and filtered three times, as described previously [20]. Cloned ureaplasmas were designated E22 5.8.1 (originating from the avirulent parent strain) and E24 3.2.1 (originating from the virulent parent strain).

### Animal model

At 55 days of gestation (d, term = 150 d) 25 pregnant Merino ewes were randomized to receive ultrasound-guided intra-amniotic injections (virulent-derived clone E24 3.2.1: n = 10; avirulent-derived clone E22 5.8.1: n = 10; 10B medium control: n = 5). Prior to the injection of ureaplasmas, AF was aspirated to verify: (i) the injection site by electrolyte analysis (Rapidlab 865, Bayer Diagnostics, Pymble, NSW); and (ii) to test that animals did not have pre-existing intra-amniotic ureaplasma infections. Ureaplasma isolates and 10B medium were prepared for injection in 2 mL volumes, which consisted of 2×10^4^ CFU or 10B medium diluted in PBS [21]. AF was sampled from each animal by ultrasound-guided amniocentesis approximately every two weeks (at 73, 87, 101, 115 and 126 days of gestation) post intra-amniotic injection and was tested by culture and western blot.

Near-term fetuses were delivered surgically at 140 d [17], [22] and samples of AF, chorioamnion, cord, fetal lung and fetal CSF were aseptically collected. Complete blood counts were performed on umbilical arterial blood.

### Ureaplasma culture

Ureaplasmas were cultured from AF, chorioamnion, cord, fetal lung and fetal CSF specimens in 10B broth medium [17], [20], [22]. Ureaplasmas within specimens were quantified by standard drop plate analysis, and are reported as the number of CFU/mL of fluid, or CFU/gram of tissue.

### *mba* PCR

To determine the size and number of *mba* variants within clonal ureaplasma isolates E22 5.8.1 and E24 3.2.1, the downstream repeat region of the *mba* was amplified by PCR, as described previously [17].

### MBA western blots

Ureaplasma MBA size variants were detected in un-cultured AF specimens collected at 73 d, 87 d, 101 d, 115 d, 126 d and 140 d by western blot. Western blots were performed directly on centrifuged AF rather than ureaplasmas cultured from AF, as culture can select for sub-populations of MBA variants, and therefore results are not representative of the pool of size variants originally present within the AF (data not shown). 10 mL of thawed AF was centrifuged at 5250× g for 20 minutes at 4°C. The supernatant was discarded and the pellet then was resuspended in 100 µL of PBS. SDS-PAGE and western blots were performed as described previously [17]. The primary antibody used for detection of the MBA was rabbit polyclonal antisera raised against *U. parvum* serovar 6 (courtesy of Emeritus Dr Patricia Quinn) diluted 1/5000 in blocking solution (5% skim milk, 150 mM NaCl, 50 mM Tris). Membranes were further probed with a goat anti-rabbit IgG-HRP secondary antibody (Sigma Aldrich, Castle Hill, NSW) diluted 1/5000; and MBA protein bands were detected by 3′, 3′-diaminobenzidine tetrahydrochloride (DAB) staining with cobalt chloride (Sigma). Proteins extracted from ureaplasma isolates E22 5.8.1 and E24 3.2.1 were included in each western blot as positive controls.

### Histopathology

Formalin-fixed paraffin-embedded tissues were cut into 5 µm sections and stained with haematoxylin and eosin (H & E, all tissue types) and Masson's trichrome stain (chorioamnion samples only). Sectioning and staining was performed by QML Vetnostics (Murarrie, QLD). Inflammatory cell counts were performed on H & E stained sections to determine the numbers of monocytes, macrophages, lymphocytes, band neutrophils and polymorphonuclear neutrophils (PMNs) in 20 fields of view at ×1000 total magnification. Masson's trichrome-stained chorioamnion sections were graded using our previously described scoring system [22] to determine the severity of histological chorioamnionitis. Histopathological analysis of H & E and Masson's trichrome stained tissues was performed blinded to animal treatment groups.

### RNA extraction and RT-PCR

Total RNA was extracted from 20 µg of freshly thawed chorioamnion and fetal lung samples collected at 140 d using the Qiagen RNeasy Mini Kit (Qiagen, Doncaster, VIC). The quantity of eluted RNA was measured using the NanoDrop 1000 spectrophotometer (Thermo Fischer Scientific Australia Pty Ltd, Scoresby, VIC) and 1.5 µg of cDNA was synthesised from RNA template by RT-PCR using Invitrogen's Super Script III First-Strand Synthesis Supermix for q-PCR (Invitrogen, Mulgrave, VIC). RT-PCR conditions consisted of: 25°C for 10 minutes, 50°C for 30 minutes, and 85°C for 5 minutes. cDNA samples were then chilled on ice, and contaminating RNA was degraded by the addition of RNase H followed by incubation at 37°C for 20 minutes. cDNA was stored at −20°C until use.

### Quantitative Real time PCR

Quantitative real time PCR was performed on chorioamnion and fetal lung cDNA samples to determine the expression of: Toll-like receptor (TLR) 1, TLR2, TLR6, interleukin (IL)-1β, IL-6, IL-8, IL-10 and tumor necrosis factor-α (TNF-α), relative to the expression of GAPDH. Previously published PCR primers [23]–[25] (Table 1) were used to amplify sheep-specific sequences. PCR assays incorporated 1× Platinum SYBR Green qPCR SuperMix-UDG (Invitrogen); either 0.4 µM of each primer (GAPDH, TLR1, TLR2 and TLR6) or 0.5 µM of each primer (IL-1β IL-6, IL-8, IL-10, TNF-α); 25 ng of cDNA and sterile distilled H_2_O to a final volume of 20 µL. Real time PCR cycling was performed in a Qiagen Rotor-Gene Q thermocycler (Qiagen, Doncaster, VIC), and included initial incubation at 50°C for 10 minutes and an initial denaturation step at 95°C for 10 minutes. Cycling then consisted of 40 cycles of: denaturation at 94°C for 15 seconds (GAPDH, TLR1, TLR2 and TLR6) or 20 seconds (IL-1β IL-6, IL-8, IL-10 and TNF-α); annealing at 52°C to 58°C (specific annealing temperatures for each primer pair are shown in Table 1); and extension at 72°C for 20 seconds (IL-1β IL-6, IL-8, IL-10 and TNF-α) or 40 seconds (GAPDH, TLR1, TLR2 and TLR6). Cycle thresholds (C_T_) were calculated using Rotor-Gene Q series software version 1.7 (Qiagen) and PCR product specificity was confirmed by standard melt curve analysis. Prior to the testing of chorioamnion and fetal lung cDNA samples, the amplification efficiency of each of the primer pairs was validated (data not shown), as per the protocol published by Schmittgen and Livak [26]. All primer efficiencies were found to be within +/−10% of the efficiency of the reference gene (GAPDH, 95% efficiency).

**Table 1:** PCR primers used for the amplification of selected Toll-like receptor (TLR) and cytokine genes from chorioamnion and fetal lung cDNA.

PRIMER	SEQUENCE (5′ to 3′)	ANNEALING TEMPERATURE	SOURCE
GAPDH (F)	GTCCGTTGTGGATCTGACCT	58°C	Chang *et al.* 2009 [24]
GAPDH (R)	TGCTGTAGCCGAATTCATTG	58°C	Chang *et al.* 2009 [24]
TLR1 (F)	TTGCACATCAGCAAGGTTTT	58°C	Chang *et al.* 2009 [24]
TLR1 (R)	CACTGTGGTGCTGACTGACA	58°C	Chang *et al.* 2009 [24]
TLR2 (F)	GGCTGTAATCAGCGTGTTCA	58°C	Chang *et al.* 2009 [24]
TLR2 (R)	GATCTCGTTGTCGGACAGGT	58°C	Chang *et al.* 2009 [24]
TLR6 (F)	TTTGTCCTCAGGAACCAAGC	58°C	Chang *et al.* 2009 [24]
TLR6 (R)	TCATATTCCAAAGAATTCCAGCTA	58°C	Chang *et al.* 2009 [24]
IL-1β (F)	CCTTGGGTATCAGGGACAA	57°C	McNeilly *et al.* 2008 [23]
IL-1β (R)	TGCGTATGGCTTTCTTTAGG	57°C	McNeilly *et al.* 2008 [23]
IL-6 (F)	TCCAGAACGAGTTTGAGG	52°C	Egan *et al.* 1996 [25]
IL-6 (R)	CATCCGAATAGCTCTCAG	52°C	Egan *et al.* 1996 [25]
IL-8 (F)	ATCAGTACAGAACTTCGA	55°C	McNeilly *et al.* 2008 [23]
IL-8 (R)	TCATGGATCTTGCTTCTC	55°C	McNeilly *et al.* 2008 [23]
IL-10 (F)	TGAAGGACCAACTGAACAGC	55°C	Egan *et al.* 1996 [25]
IL-10 (R)	TTCACGTGCTCCTTGATGTC	55°C	Egan *et al.* 1996 [25]
TNF-α (F)	GAATACCTGGACTATGCCGA	58°C	McNeilly *et al.* 2008 [23]
TNF-α (R)	CCTCACTTCCCTACATCCCT	58°C	McNeilly *et al.* 2008 [23]

IL = interleukin, TNF = tumor necrosis factor, F = forward primer, R = reverse primer.

All chorioamnion and fetal lung samples were tested in triplicate and mean C_T_ values from animals infected with virulent-derived (E24 3.2.1) and avirulent-derived (E22 5.8.1) ureaplasmas were used to calculate the expression of target genes relative to GAPDH and normalised against the expression of target genes in non-infected control animals. Relative expression was determined by the equation published by Pfaffl [27], where the relative expression ratio = (Efficiency_target gene_)^ΔCT(control – sample)^/(Efficiency_reference gene_)^ΔCT(control – sample)^. This method was selected as opposed to the ΔΔC_T_ method of relative expression, as the equation presented by Pfaffl [27] does not assume that PCR efficiencies are equal between the reference and target genes.

### Detection of anti-ureaplasma IgG antibodies

Western blots were performed to determine if anti-ureaplasma IgG antibodies were present in maternal and/or fetal sera, as indicators of a humoral immune response. Standardised protein extracts of whole ureaplasma isolates E22 5.8.1 and E24 3.2.1 were loaded into the wells of 10% SDS-PAGE gels, electrophoresed, transferred onto nitrocellulose membrane and blocked as described above. Maternal or fetal serum (diluted 1/100 in blocking solution) was used as the primary antibody, and membranes were incubated with these sera overnight at 4°C. Membranes were washed and then probed with secondary antibody (anti-sheep IgG (whole molecule)-HRP, raised in donkey (Sigma)) diluted 1/1000. The presence of protein bands (detected by DAB staining with cobalt chloride (Sigma)) indicated binding of antibodies within the serum to ureaplasmal proteins from either the virulent-derived or avirulent-derived ureaplasma strain. All samples were tested in duplicate.

### Serial passage of virulent and avirulent-derived clonal ureaplasmas

Serial passage experiments were performed by inoculating 2×10^4^ CFU/mL of ureaplasma strains E22 5.8.1 and E24 3.2.1 into 1.8 mL of 10B medium containing rabbit polyclonal *U. parvum* serovar 6 antiserum (at a final dilution of 1/500). Inoculated broths were incubated at 37°C aerobically until a colour change was evident within the media (usually occurring between 12 and 18 hours). Ureaplasmas were then transferred into fresh 10B medium (at a concentration of approximately 10^4^ to 10^5^ colour changing units per mL) containing antibodies, and were again incubated. Each ureaplasma isolate was serially passaged 20 times in culture media containing antibodies. After each passage, samples were collected for western blot. As a control, ureaplasma isolates E22 5.8.1 and E24 3.2.1 were also serially transferred in 10B medium without antibodies 20 times.

### Statistical analysis

Pregnancy outcomes, complete blood count data, inflammatory cell counts and ureaplasma tissue colonization data were initially analysed for homogeneity of variances by Levene's test. Those data for which homogeneity of variances were confirmed were subsequently analysed by one-way analysis of variance (ANOVA) with a Tukey post hoc test. If the assumption of homogeneity of variance was violated, the Welch statistic was alternatively reported. Fetal lung compliance, AF colonization and MBA size variant data were analysed by a two-way repeated measures ANOVA, and degrees of freedom were corrected using Greenhouse-Geisser estimates if the assumption of sphericity was violated. Independent t-tests were used to analyse humoral immune response and qRT-PCR data. Data are presented as mean ± standard error of the mean (SEM) and statistical significance was accepted at p<0.05.

## Results

### Pregnancy outcomes

Of the 25 pregnant ewes that received intra-amniotic injections, fetuses were spontaneously aborted from three ewes (virulent group n = 2; avirulent group n = 1) at approximately 82 d, 115 d and 131 d (Table 2). One ewe (avirulent group) also delivered a stillborn fetus at 131 d after preterm labor. Oligohydramnios was observed at least once during the amniocentesis sampling period in three animals from the avirulent group, in two animals from the virulent group, but not in the control group. Meconium-stained AF was present in four animals from the virulent group at least once throughout the sampling period; however, meconium was not present in AF from animals from the avirulent group or the media control group. Pregnancy loss and oligohydramnios occurred independently of the animal group (p = 0.59 and p = 0.42 respectively). The presence of meconium-stained AF was significantly increased in the virulent group compared to the avirulent group and the media control group (p = 0.01).

**Table 2:** Pregnancy outcomes and fetal measurements at the time of delivery (140 d).

	VIRULENT GROUP	AVIRULENT GROUP	CONTROL GROUP	P VALUE
***Pregnancy outcomes***				
Abortion/stillborn fetus	2 (20%)	2 (20%)	0 (0%)	0.59
Oligohydramnios	2 (20%)	3 (30%)	0 (0%)	0.42
Meconium-stained amniotic fluid	4 (40%)	0 (0%)	0 (0%)	0.01
Gender (female ∶ male)	5∶3	5∶3	3∶2	0.10
Fetal birth weight (kg)	4.8±0.3	5.3±0.2	5.3±0.3	0.36
Fetal lung weight (g/kg body weight)	28.3±1.4	31.4±1.9	30.4±2.8	0.50
Lung volume (mL/kg) at 40 cm H_2_O pressure	36.2±4.0	42.1±1.7	37.7±3.6	0.06
***Umbilical arterial cord blood gases***				
pH	7.2±0.03	7.21±0.03	7.19±0.05	0.09
pO_2_ (mmHg)	9.6±0.6	9.9±0.9	12.8±1.6	0.08
***Umbilical arterial white blood cell counts***				
Total (×10^9^/L)	4.2±0.9	3.9±0.5	3.6±0.6	0.87
Monocytes (×10^9^/L)	0.3±0.1	0.1±0.04	0.2±0.1	0.54
Lymphocytes (×10^9^/L)	2.2±0.3	2.0±0.2	2.1±0.4	0.86
Neutrophils (×10^9^/L)	1.2±0.6	0.9±0.1	0.8±0.2	0.75

Fetal birth weight, fetal lung weight and umbilical arterial cord blood pH, pO_2_ and white blood cell counts were not different between animal groups (p>0.05, Table 2). Chronic intra-amniotic infection with the avirulent-derived (E22 5.8.1) ureaplasma strain tended to increase lung compliance in near-term fetuses (as determined by a deflation pressure-volume curve, Table 2), when compared to the virulent and control groups. However this observed increase was not statistically significant (p = 0.06).

### Ureaplasmas can chronically colonise the amniotic fluid

**Figure 1:**
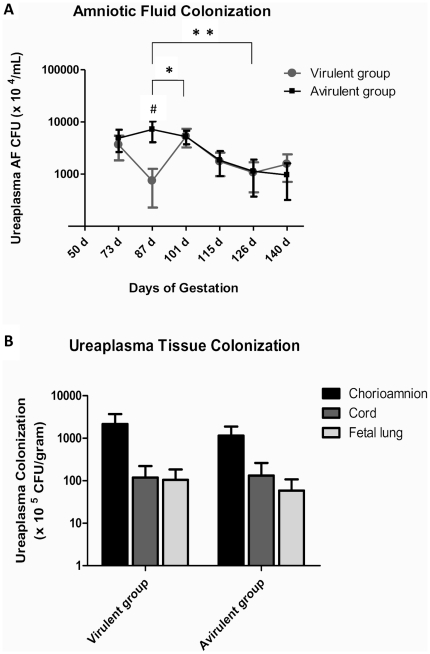
Ureaplasma colonization of amniotic fluid and fetal tissues. (**A**) Chronic infection of the amniotic fluid was observed in all ewes experimentally infected with ureaplasmas from the time of inoculation (55 d) until fetuses were delivered at 140 d. Amniotic fluid ureaplasma colonization was significantly increased in the avirulent group when compared to the virulent group at 87 d (p<0.05, denoted by #). Statistically significant differences in amniotic fluid ureaplasma colonization within animal groups occurred between 87 d and 101 d; and 87 d and 126 d (p<0.05). (**B**) Ureaplasmas were isolated from the chorioamnion, cord and fetal lung; however, recovered ureaplasma CFU/g was not different between animal groups for the tested tissue types. * = statistically significant difference between time points in the virulent group only; ** = statistically significant difference between time points in both groups. AF = amniotic fluid; CFU = colony forming units; d = days of gestation. Data are presented as mean ± SEM.

AF collected from all ewes prior to intra-amniotic injection at 55 d tested negative for ureaplasmas. Following intra-amniotic injection, the AF from all ewes inoculated with ureaplasmas (either isolate E22 5.8.1 or isolate E24 3.2.1) tested positive for ureaplasmas at all time points (Figure 1A). The peak of infection occurred at 87 d for the avirulent group (7.2±3.1×10^7^ CFU/mL) and at 101 d for the virulent group (5.3±2.1×10^7^ CFU/mL). Ureaplasma AF colonization at 87 d was significantly increased in the avirulent group compared to the virulent group (p = 0.002); however, there were no differences in colonization at any other time points between these groups. High numbers of ureaplasmas were recovered from the AF in both the avirulent (9.6±6.4×10^6^ CFU/mL) and virulent (1.6±0.8×10^7^ CFU/mL) groups at 140 d. For each experimental animal group differences in the AF ureaplasma CFU/mL were observed: (i) between 87 d and 101 d for the virulent group only (p = 0.04); and (ii) between 87 d and 126 d for both the avirulent and virulent groups (p = 0.02). Ureaplasmas were not detected in the AF of non-infected controls.

All animals from the avirulent and virulent groups tested culture-positive for ureaplasmas within the chorioamnion at 140 d (Figure 1B). Ureaplasmas were cultured from the cords of 4 out of 8 (50%) animals from both the virulent and avirulent groups; and from the fetal lungs of 5 out of 8 (62.5%) lambs from the virulent group and 6 out of 8 (75%) lambs from the avirulent group. No ureaplasmas were detected in fetal CSF specimens (as determined by both culture and PCR, data not shown), or from any tissue specimens from non-infected control animals. Ureaplasma colonization in chorioamnion (p = 0.38), cord (p = 0.66) and fetal lung (p = 0.49) tissues were not different between treatment groups.

### Intra-amniotic ureaplasma infection is associated with fetal inflammation

**Figure 2:**
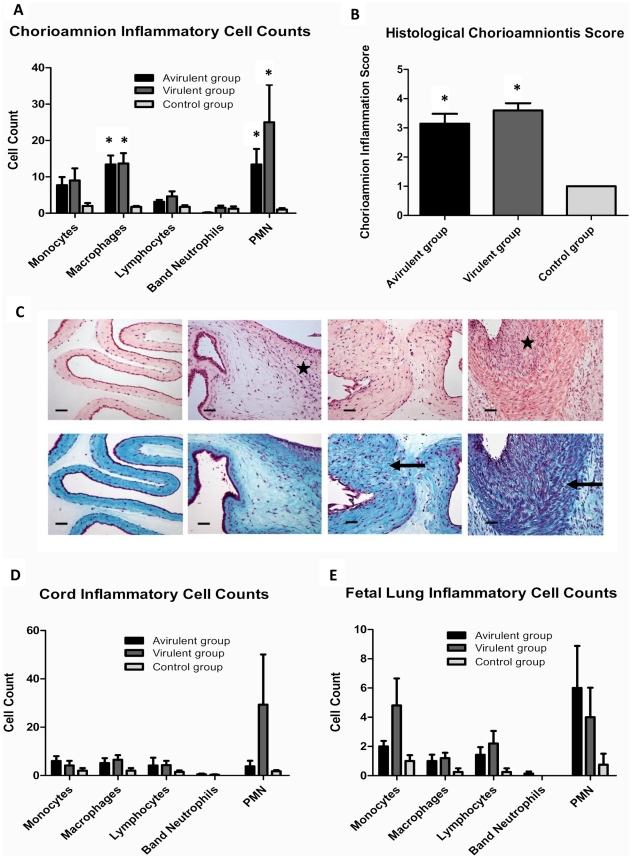
Fetal inflammation induced by intra-amniotic ureaplasma infection. Inflammatory cell infiltrates within chorioamnion tissue (**A**) and the severity of histological chorioamnionitis (**B**) were increased in animals from the virulent and avirulent groups when compared to the control group. Representative chorioamnion sections ((**C**) stained with haematoxylin and eosin (top row) or Masson's trichrome stain (bottom row), photographed at ×200 total magnification) demonstrate the 4 stages of histological chorioamnionitis. From left to right: Grade 1 (uninfected control), minimal inflammatory cell infiltrate and no tissue fibrosis, necrosis or abscesses; Grade 2, mild inflammatory cell infiltrate and mild tissue fibrosis, necrosis or abscesses; Grade 3, heavy inflammatory cell infiltrate and moderate tissue fibrosis, necrosis or abscesses; Grade 4, heavy inflammatory cell infiltrate and sever fibrosis, necrosis or abscesses. Stars on haematoxylin and eosin stained sections indicate localized inflammatory cell influx. Arrows on Masson's trichrome stained sections represent tissue fibrosis and disruption of normal tissue morphology. Size bars represent 50 µm. Inflammatory cell infiltrates within cord tissue (**D**) and fetal lung tissue (**E**) were not statistically different between treatment groups. Data are presented as mean + SEM. * p<0.05 when compared to the control group.

Inflammatory cell counts within chorioamnion tissue were higher in animals injected with virulent-derived and avirulent-derived ureaplasmas when compared to controls. However, inflammatory cell counts were not different between the virulent and avirulent groups for any of the cell types (p>0.05, Figure 2A). The number of macrophages and PMNs within chorioamnion sections were significantly increased in both the virulent and avirulent groups when compared to controls (p = 0.02 and p = 0.03 respectively). Grading of chorioamnion sections demonstrated that animals in the 10B medium control group had no evidence of histological chorioamnionitis, but moderate to severe histological chorioamnionitis was evident in both the avirulent and virulent groups (p = 0.001, Figure 2B). Representative chorioamnion sections stained with H & E and Masson's trichrome stain (Figure 2C) demonstrated the various grades of histological chorioamnionitis. Non-infected chorioamnion tissues were characterized by minimal/no inflammatory cell influx and a well-defined structure consisting of a thin layer of fibroblasts bordering intact epithelial cells. In contrast, histological chorioamnionitis was associated with increased localized inflammatory cell influx, fibrosis and/or scar tissue (as indicated by thickening of fibroblast layers and lesion formation) and an irregular epithelial layer. Umbilical cord and fetal lung inflammatory cell counts were not different between groups (Figures 2D and 2E, p>0.05).

### MBA size variation occurs *in vivo*

**Figure 3:**
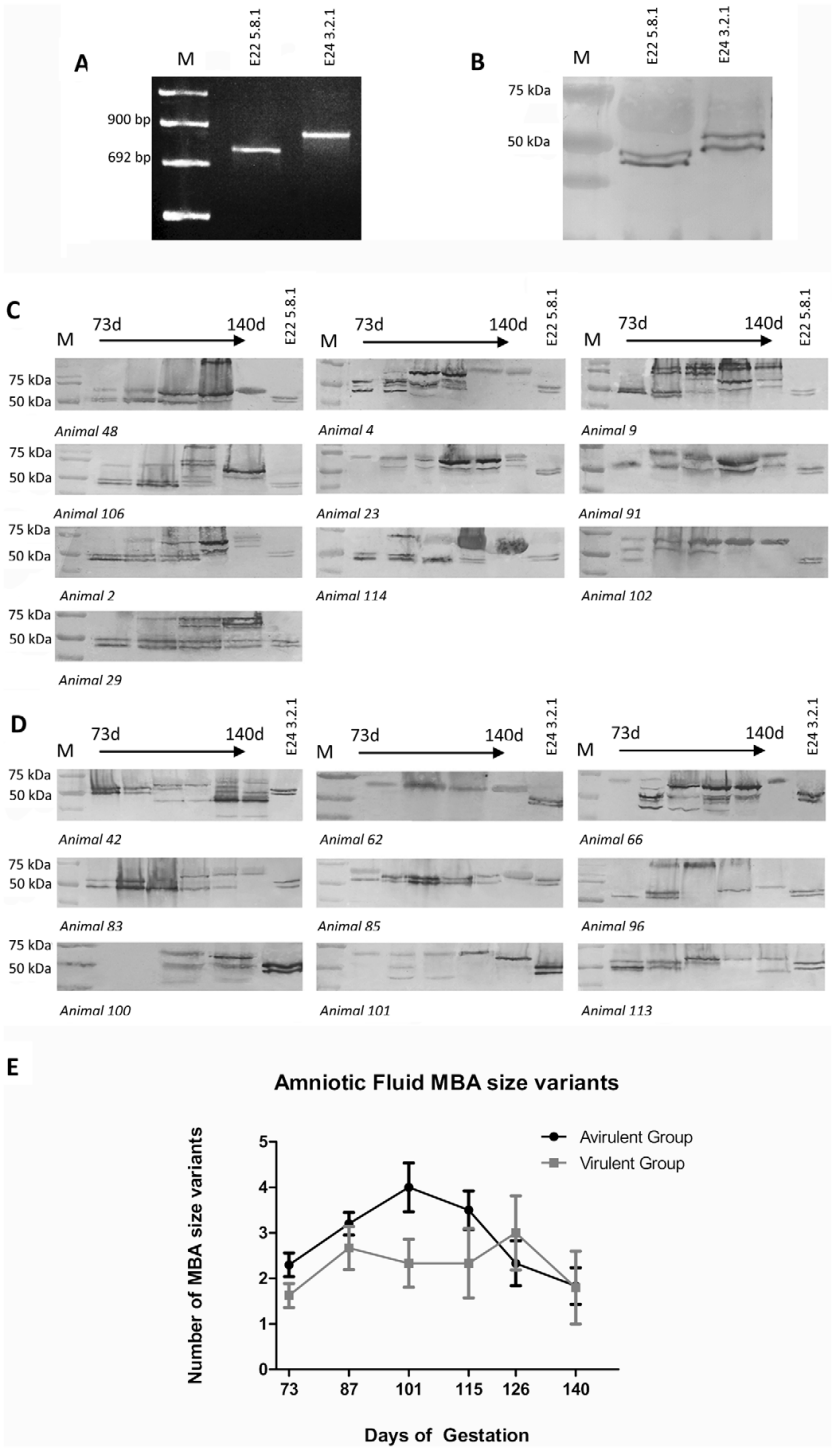
Size variation of the MBA was observed in amniotic fluid samples. PCR of the repeat region of the *mba* from ureaplasma isolates E22 5.8.1 and E24 3.2.1 produced single amplicons prior to injection into pregnant sheep (**A**), indicating that these clonal isolates contained only one *mba* size variant; however, western blots of the same isolates (**B**) demonstrated that the MBA appears as a double band. MBA size variants were detected *in vivo* within the amniotic fluid of animals from the avirulent group (**C**) and the virulent group (**D**) by western blot. Each western blot demonstrates the ureaplasma MBA size variants generated at 73, 87, 101, 115, 126 and 140 days of gestation in each animal. Note: samples were not collected at some time points for certain animals due to oligohydramnios or other complicating factors. Protein preparations from the avirulent-derived (E22 5.8.1) and virulent-derived (E24 3.2.1) ureaplasma clones used for intra-amniotic injection were included in each western blot (in the last lane) as a positive control and for size comparison. M = DNA molecular weight marker VIII (Roche, Castle Hill, New South Wales) or protein marker (BioRad, Gladesville, New South Wales). The average number of MBA size variants generated over time (**E**) was not different between animal groups.

Ureaplasma MBA size variants were detected by western blot in AF specimens collected at 73 d, 87 d, 101 d, 115 d, 126 d and 140 d. The avirulent-derived and virulent-derived ureaplasma clones used for intra-amniotic injection each expressed a single *mba* size variant as determined by PCR (Figure 3A); however, these appeared as double bands in western blots (Figure 3B). MBA bands of approximately 45 kDa and 50 kDa (E22 5.8.1); and 50 kDa and 55 kDa (E24 3.2.1) were detected in the avirulent-derived and virulent-derived strains respectively. Antigenic size variation was detected within all tested AF samples from the avirulent group (Figure 3C) and the virulent group (Figure 3D), and MBA bands ranged in size from 32 kDa to 170 kDa. In the avirulent group all detected MBA proteins were equal in size to, or had an increased molecular weight when compared to the E22 5.8.1 inoculum. In the virulent group, the range of MBA size variants was greater, as MBA bands of lower, equal and higher molecular weight were detected.

From these AF specimens, the total numbers of MBA size variants detected ranged from one to eight. The average number of AF MBA size variants in the avirulent group increased over the first three time points and reached a maximum of four variants at 101 d (Figure 3E). Following this, the number of MBA size variants decreased over the last three time points. Within the virulent group the average number of MBA size variants within the AF initially increased at 87 d, peaked at 126 d, and then decreased at 140 d. The number of MBA size variants detected within AF specimens was not different between animal groups at any of the tested time points (p = 0.87), nor were there differences in the number of MBA size variants within the two groups for the entire gestation (p = 0.32).

### Intra-amniotic ureaplasma infection stimulates a maternal and fetal humoral response

**Figure 4:**
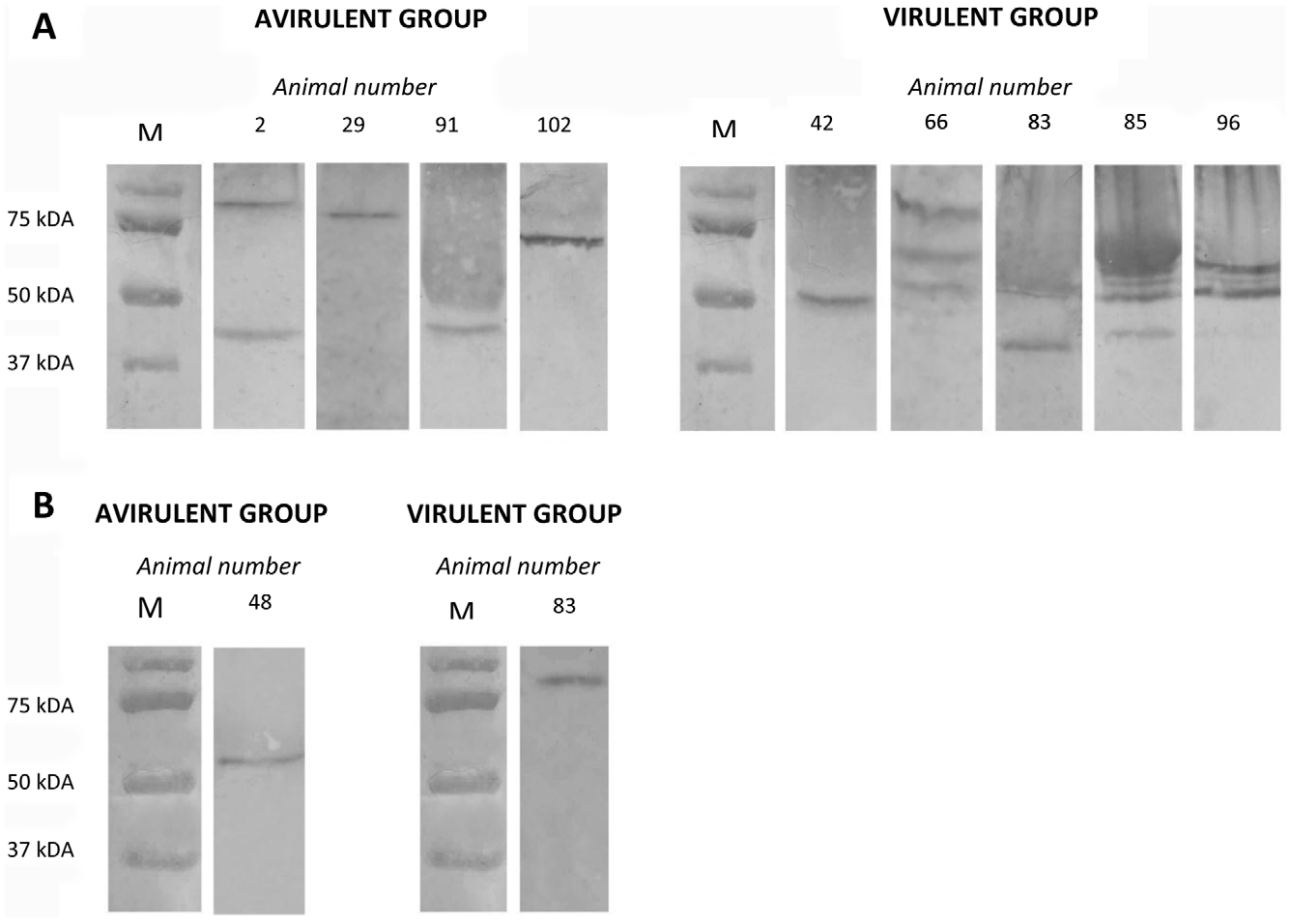
Demonstration of maternal and fetal anti-ureaplasma humoral responses. Anti-ureaplasma IgG antibodies were detected in the maternal serum (**A**) and the fetal serum (**B**) of animals in both the avirulent and virulent groups. Antibodies in serum samples reacted with ≥1 ureaplasmal protein, over a wide molecular weight range (approximately 45 kDa to 80 kDa). Numbers above western blots indicate the animal number from which the serum was obtained. M = protein marker (BioRad).

Anti-ureaplasma IgG antibodies were detected in the maternal serum of four ewes from the avirulent group and five ewes from the virulent group (Figure 4A). In the avirulent group, the maternal antibodies from each positive ewe reacted with ureaplasmal proteins (ranging in size from approximately 45 kDa to 87 kDa), which were different in size to the MBA variants detected in AF specimens (as determined by molecular weight comparison). Within this avirulent group, the maternal serum of one ewe (animal 2) reacted with two ureaplasmal proteins, whilst the sera from the other three animals reacted with only one protein. Positive sera were also probed against protein extracts from MBA-negative avirulent-derived ureaplasma clones (generated from serial *in vitro* transfer experiments). Comparison of protein bands recognised by maternal serum when probed against whole ureaplasma protein extract or MBA-negative ureaplasma protein extract (Table 3) demonstrated that two (out of four) serum samples from the avirulent group detected a protein of approximately 45 kDa, which was expressed in both whole ureaplasma and MBA-negative ureaplasma preparations.

**Table 3:** Comparison of the protein bands detected by anti-ureaplasma IgG antibodies in maternal serum when probed against whole ureaplasma protein extract or MBA-negative ureaplasma protein extract.

		PROTEIN BANDS RECOGNISED BY MATERNAL SERUM WHEN PROBED AGAINST:	
ANIMAL #	TREATMENT GROUP	Whole ureaplasma protein extract	MBA-negative ureaplasma protein extract
2	Avirulent	**45 kDa**, 87 kDa	**45 kDa**
29	Avirulent	80 kDa	N/A
91	Avirulent	**45 kDa**	**45 kDa**
102	Avirulent	70 kDa	N/A
42	Virulent	50 kDa	45 kDa
66	Virulent	55 kDa, 62 kDa, 87 kDa	N/A
83	Virulent	**45 kDa**, 50 kDa	**45 kDa**
85	Virulent	45 kDa, 50 kDa, 55 kDa, 60 kDa	N/A
96	Virulent	50 kDa, 55 kDa, 60 kDa	45 kDa

Those proteins indicated in bold were detected in western blots using both whole ureaplasma and MBA negative ureaplasma protein extracts. kDa = kilodaltons.

In the virulent group, one ewe (animal 42) produced antibodies only against a 50 kDa protein, whereas the other four ewes produced antibodies against more than one ureaplasmal protein (ranging in size from approximately 45 kDa to 80 kDa), including (but not limited to) MBA size variants that were detected within the AF of each animal. When positive sera from the virulent group were probed against MBA-negative virulent-derived ureaplasma protein extracts, three (out of five) serum samples also detected a protein of approximately 45 kDa (Table 3).

Maternal serum collected from non-infected controls did not react with ureaplasmal proteins and 10B medium, demonstrating that antibodies were not generated against ureaplasmas or 10B medium components. The number of ewes which generated anti-ureaplasma IgG antibodies was not different between the avirulent and virulent groups (p = 0.35).

Anti-ureaplasma IgG was present within the fetal serum of one fetus delivered from the avirulent group, and one fetus delivered from the virulent group (Figure 4B). Fetal serum from both positive fetuses reacted with only one ureaplasmal protein (animal 48, avirulent group: 60 kDa; and animal 83, virulent group: 80 kDa), both of which were not detected within the pool of MBA size variants from the corresponding AF sample. Interestingly, anti-ureaplasma IgG antibodies were produced in both the maternal and fetal serum of animal 83; however, these sera reacted with different ureaplasmal proteins (maternal serum: approximately 45 kDa and 55 kDa; fetal serum: approximately 80 kDa).

### Relative expression of ovine Toll-like receptors and cytokines

Within chorioamnion tissue there were no statistically significant increases or decreases in the expression of ovine Toll-like receptors or cytokines between the avirulent and virulent groups (Table 4). The expression of IL-1β, IL-6 and IL-8 tended to be up-regulated in both the avirulent and virulent groups, relative to the expression of GAPDH, although high levels of intra-animal variation were observed, suggesting significant variability in the host immune response. Within both ureaplasma-infected groups, the relative expression levels of TLR1, TLR2 and TLR6 were similar to expression levels in media control animals, whereas the expression of TNF-α and IL-10 were slightly down-regulated (albeit not statistically significant).

**Table 4:** The relative expression of ovine Toll-like receptors and cytokines in chorioamnion and fetal lung tissue, relative to GAPDH.

	CHORIOAMNION			FETAL LUNG		
	AVIRULENT GROUP	VIRULENT GROUP	P VALUE	AVIRULENT GROUP	VIRULENT GROUP	P VALUE
**TLR1**	−2.0±3.1	−2.7±1.9	0.9	0.2±0.7	12.3±10.8	0.3
**TLR2**	−3.1±2.9	−3.1±2.9	0.9	−2.7±0.4	12.3±14.6	0.3
**TLR6**	−3.1±2.6	−2.8±1.7	0.9	−0.5±0.5	18.3±17.4	0.3
**IL-1β**	5.4±2.9	48.2±45.5	0.4	−13.2±3.7	−9.2±11.9	0.7
**TNF-α**	−7.4±4.9	−4.0±3.3	0.6	−0.7±0.7	81.3±81.1	0.4
**IL-6**	47.5±44.1	71.8±39.7	0.7	−30.9±12.8	−7.6±9.3	0.2
**IL-8**	87.3±71.4	98.6±81.4	0.9	−38.3±11.6	−32.1± 2.2	0.8
**IL-10**	−5.8±4.3	−8.5±5.9	0.7	−1.8±0.7	230.6±229.5	0.4

Data are presented as mean ± SEM. TLR = Toll like receptor, IL = interleukin, TNF = tumor necrosis factor.

Similarly, within fetal lung tissue, high levels of intra-animal variation were observed and there were no statistically significant differences in the expression of Toll-like receptors or cytokines between the avirulent and virulent groups (Table 4). Within the avirulent group, the expression of IL-1β, IL-6 and IL-8 tended to be down-regulated relative to GAPDH, whereas in the virulent group some animals demonstrated up-regulation of TNF-α and IL-10.

**Figure 5:**
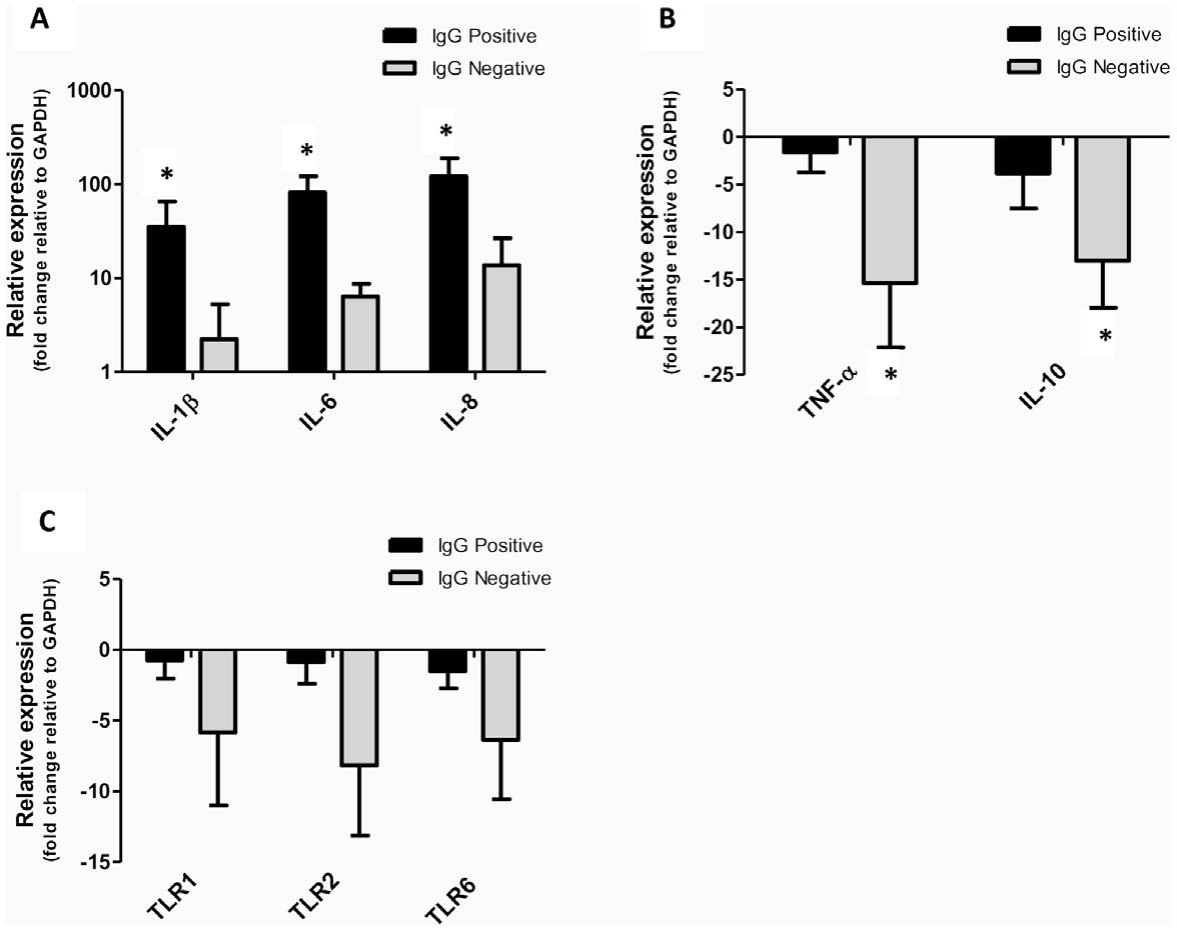
Pro-inflammatory cytokines were up-regulated in animals which produced ureaplasma-specific ureaplasma antibodies. The relative expression of IL-1β, IL-6 and IL-8 within the chorioamnion was significantly increased in ewes that tested positive for anti-ureaplasma IgG antibodies (IgG positive) within serum samples, when compared to animals in which these antibodies were not generated (IgG negative) (**A**). Conversely, the relative expression of TNF-α and IL-10 (**B**); and TLR1, TLR2 and TLR6 (**C**) within the chorioamnion were decreased in IgG negative animals when compared to IgG positive animals. Data are presented as mean fold change ± SEM. * p<0.05. Expression of genes is determined relative to the expression of GAPDH after normalisation against 10B medium control animals.

Interestingly, when these gene expression data were grouped based on the presence of anti-ureaplasma IgG antibodies within maternal serum (as opposed to treatment group) significant differences were found. Specifically, the relative expression of IL-1β, IL-6 and IL-8 within the chorioamnion was significantly increased in animals which tested positive for anti-ureaplasma IgG antibodies, when compared to those animals which tested negative for the presence of these antibodies (IL-1β: p = 0.04; IL-6: p = 0.02; IL-8: p = 0.04, Figure 5A). Furthermore, the relative expression of TNF-α (p = 0.02) and IL-10 (p = 0.04) within chorioamnion tissue was significantly decreased in IgG negative animals, when compared to those animals which were positive for anti-ureaplasma IgG antibodies (Figure 5B). The relative expression of TLR1, TLR2 and TLR6 also tended to be decreased in IgG negative animals, when compared to IgG positive animals; however, these data were not statistically significant (p = 0.20, p = 0.08, p = 0.16 respectively, Figure 5C).

### Serial passage of virulent and avirulent ureaplasmas

**Figure 6:**
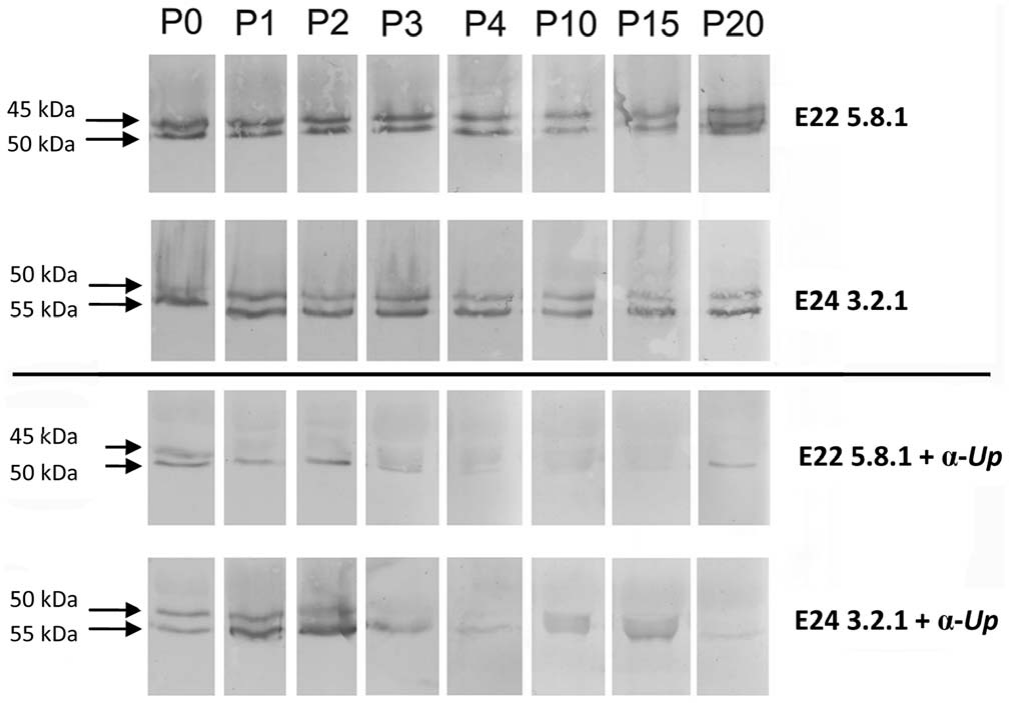
Ureaplasmas are phase variable *in vitro*. Western blot analysis demonstrated that MBA expression was not affected in avirulent-derived (E22 5.8.1) and virulent-derived (E24 3.2.1) ureaplasmas, which were serially transferred in 10B medium without the presence of polyclonal antibodies (top panel). MBA negative escape variants were generated for both avirulent-derived and virulent-derived ureaplasma strains after serial transfer in 10B medium containing anti-ureaplasma polyclonal antibodies (α-*Up*, bottom panel). P = passage number.

Serial passage of avirulent-derived E22 5.8.1 and virulent-derived E24 3.2.1 ureaplasma strains in 10B medium without the presence of anti-ureaplasma polyclonal rabbit sera did not lead to the emergence of MBA escape variants or MBA size variants after 20 passages (Figure 6). Conversely, when these strains were serially transferred in 10B medium containing rabbit anti-ureaplasma antibodies, MBA escape variants were generated in both the avirulent-derived and virulent-derived ureaplasma isolates (Figure 6). MBA-negative ureaplasmas were generated after three serial transfers for isolate E22 5.8.1, and after four serial transfers for isolate E24 3.2.1. MBA size variation was not observed in any of these isolates.

## Discussion

The pathogenic role of *Ureaplasma* spp. in adverse pregnancy outcomes is controversial. The isolation of these microorganisms from the upper genital tract of pregnant women who deliver at term with no evidence of chorioamnionitis [8], [9], [11] suggests that a causal relationship between intra-amniotic ureaplasma infection and adverse pregnancy outcomes does not always exist. However, advances in our understanding of the complexities of disease pathogenesis have changed how we define microbial pathogens and highlighted the importance of host-pathogen interactions in predicting disease [28]. In this study, we investigated the role of the MBA from virulent-derived and avirulent-derived ureaplasma strains in a fetal sheep model of chronic intra-amniotic infection. Our data suggest that ureaplasmas are not intrinsically virulent/avirulent, as size variation of the MBA did not directly contribute to fetal inflammation and chorioamnionitis. However, variation of this surface-exposed antigen may prevent the eradication of ureaplasmas by the host immune response. For the first time, we have demonstrated a significant association between the up-regulation of chorioamnion pro-inflammatory cytokines and the presence of maternal serum anti-ureaplasma antibodies. We predict that a strong host immune response may be an important determinant in distinguishing asymptomatic intra-amniotic ureaplasma infections from those resulting in adverse outcome.

Our data demonstrate that the incidences of fetal abortion and oligohydramnios were not different between animals that received intra-amniotic injections of virulent-derived ureaplasmas, avirulent-derived ureaplasmas or 10B medium. Lung compliance appeared to be increased in the avirulent group when compared to the virulent and control groups; however, the number of animals studied was too few to demonstrate statistical significance. Previous data published by our group showed that long term intra-amniotic ureaplasma infection induced fetal lung compliance in preterm lambs (125 d) when compared to controls [29], [30]. However, fetuses in the current experiment were delivered at 140 d (near-term), at which time fetal lungs are in the late saccular/early alveolar developmental phase [31] and are mature. We observed a significant increase in the presence of meconium-stained AF in the virulent group compared to both the avirulent and control groups. Meconium-stained AF is reported to be a sign of fetal stress and was associated previously with increased incidences of intra-amniotic infection, chorioamnionitis and intraventricular haemorrhage [32]–[36]. However, in our experiment fetal outcomes were not different between animals with/without meconium-stained AF, suggesting that the presence of meconium within the AF may be an indirect indicator of infection and/or fetal distress in some animals.

To the best of our knowledge, this is the first study to demonstrate the progress of chronic intra-amniotic ureaplasma infections over time. Remarkably, after 85 days of *in utero* infection, ureaplasma colonization within the AF remained elevated throughout gestation, as determined by amniocentesis sampling at two weekly intervals. Even at 140 d, the AF ureaplasma CFU/mL in both the avirulent and virulent animal groups was still two-to-three logs higher than the original inoculum dose of 2×10^4^ CFU, demonstrating that AF is an excellent growth medium for these microorganisms. It is well documented that AF has bacteriostatic/bacteriocidal activity against numerous bacteria such as *Listeria monocytogenes*, *Escherichia coli*, *Staphylococcus aureus* and group B streptococci [37]–[39] due to various components within the AF including zinc and phosphate [40], [41]. Therefore, the ability of the ureaplasmas to thrive within the AF suggests that this may be a niche environment for these microorganisms.

Our results indicate that ureaplasma colonization of the AF was not affected by treatment group, as there were no differences in CFU/mL (except at 87 d) between the avirulent and virulent animal groups. Previously, we demonstrated that ureaplasma colonization of the AF and fetal tissues occurred independently of the serovar as well as the dose (high dose or low dose) used for intra-amniotic injection [17]. We predict that a major regulator of ureaplasmal growth within the AF may be pH, as ureaplasmas require a pH of 6.0 for optimal growth [42] and the pH of ovine AF ranges from 8.4 (at 70 d) to 7.4 (at 145 d) [43]. Similarly in humans, the pH of AF from third trimester pregnancies is usually 7.1±0.08 [44], which may have a limiting effect on ureaplasmal growth. Long-term colonization of the AF may be facilitated by biofilm formation at the chorioamnion-AF interface, or within ‘amniotic fluid sludge’ (as reported by Romero *et al.* [45]). Garcia-Castillo *et al.* [46] demonstrated that 82% of ureaplasmas isolated from urine specimens collected from males diagnosed with urethritis, chronic prostatitis or healthy individuals formed biofilms *in vitro*. We also have preliminary data to suggest that ureaplasmas isolated from the chorioamnion of experimentally-infected sheep are capable of forming biofilms (Dando *et al.* 2010, unpublished), indicating that this may be an important survival mechanism for these microorganisms *in vivo*.

We did not observe differences in the severity of histological chorioamnionitis between animals infected with the avirulent-derived ureaplasma clone or the virulent-derived ureaplasma clone. Both ureaplasma-infected groups had moderate to severe chorioamnionitis, as determined by tissue scoring and inflammatory cell counts. This is in contrast with our previous findings, in which infection with the parent strains of avirulent (E22 5.8.1) and virulent (E24 3.2.1) clones resulted in either no histological evidence of chorioamnionitis, or severe chorioamnionitis respectively [17]. We hypothesise that these differences may be attributed to the elaboration of different MBA size variants *in utero* between these two studies. In this experiment, both the avirulent-derived and virulent-derived ureaplasma clones produced low numbers of MBA variants *in vivo* (average = 4.2 and 4.6 size variants respectively). Whereas previously, intra-amniotic infection with a clinical ureaplasma isolate elaborated the avirulent parent strain (associated with 14 MBA variants) and the virulent parent strain (associated with 5 MBA variants). Despite differences in the numbers of MBA size variants generated between these two studies, the present data do confirm our previous observation that low numbers of MBA size variants (≤5) are associated with severe histological chorioamnionitis.

At the present time, we do not know the mechanisms that drive MBA size variation *in vivo*, and are unable to explain the differences in the number of MBA variants produced by the clonal strains in comparison to the parent strains. However, an important distinction between these two studies is that the inoculum strain used for the previous experiment [17] was a non-clonal clinical ureaplasma isolate, which may have comprised a mixture of MBA subtypes. In contrast, the inocula used for the current experiment were clonal strains, each expressing a single *mba* variant. Our data demonstrate that clonal ureaplasma strains, unlike non-clonal mixtures, may have a limited ability to generate MBA size variants in *vivo*. Furthermore, clonal selection based on MBA antigenic variation may have also selected for clones with altered expression of other proposed ureaplasmal virulence factors, which include urease, and phospholipase A and C [3]. Therefore, experiments to characterize the expression of these additional virulence factors throughout gestation may elucidate differences between clonally-derived ureaplasma strains.

Antigenic variation, as defined by Deitsch *et al.* [47], refers to the capacity of a microorganism to alter the proteins exposed to the host immune system, such that the host is confronted with a continually changing antigenic population that is difficult to eliminate. Antigenic variation can refer to either phase variation (on/off switching) or expression of alternate forms of an antigen (such as size variation). Perhaps the best characterised examples of antigenic variation are flagella phase variation in *Salmonella* spp. [48] and Opa phase variation in *Neisseria* spp. [49]. However, high frequency antigenic variation is also prominent in numerous *Mycoplasma* spp. To date, the mechanisms of antigenic variation described in mycoplasmas include: (i) slipped strand mispairing and/or nucleotide insertions/deletions in simple sequence repeats; and (ii) DNA rearrangements via site specific recombination and promoter inversions [50]. Zimmerman *et al.* [16] demonstrated that the MBA of *U. parvum* underwent alternate phase variation with an adjacent gene, UU376. By *in vitro* selection using antibody pressure, it was demonstrated that alternate expression of the MBA/UU376 was associated with a DNA inversion event in which the 5′ conserved region of the *mba* and its putative promoter were opposed to either the 3′ repeat region of the *mba* or UU376. Furthermore, phase variation of MBA N-terminal paralogs (UU171 and UU172) was recently described in both *U. parvum* and *U. urealyticum* [51]. Similarly to the alternate expression of MBA/UU376, phase variation of UU171/UU172 is predicted to occur via DNA inversion and rearrangement of potential promoter sequences.

Whilst Zimmerman *et al.* [16] demonstrated that *in vitro* antibody selection led to the emergence of MBA-negative escape variants (via phase variation), our *in vivo* data demonstrated size variability of the MBA after injection of clonal ureaplasmas expressing a single *mba* variant. PCR of the repeat region of the *mba* confirmed that isolates E22 5.8.1 and E24 3.2.1 each had one *mba* size variant prior to intra-amniotic injection; however, the MBA appeared as a double band in western blots. These findings are similar to those reported by Zheng *et al.* [14], who suggested that MBA doublets may be due to inefficient signal peptide cleavage. However, it is also possible that this may be a result of post-translational modification, such as glycosylation, but this remains to be confirmed.

We have previously reported that ureaplasmas isolated from the AF of pregnant sheep undergo MBA size variation [17]. As yet, MBA-negative ureaplasmas have not been isolated from patients or generated *in vivo*, therefore it is possible that phase variation of the MBA is induced only by strong selection using antibodies directed against the repetitive region of the MBA. To select for MBA-negative escape variants, Zimmerman *et al.* [16] incubated low numbers of clonal ureaplasmas with hyperimmune antisera diluted 1/100. It is highly unlikely that these conditions would be mimicked *in vivo*, which is perhaps why we have not observed MBA phase variation in our sheep model. To determine whether isolates E22 5.8.1 and E24 3.2.1 were capable of MBA phase variation, we applied antibody pressure to these clonal ureaplasmas *in vitro* and found that MBA-negative variants were elaborated after either three or four serial transfers. Similarly, we were only able to achieve this using a high concentration of rabbit antisera, which further suggests that MBA phase variation may only be inducible *in vitro* using concentrated antibodies.

A primary function of antigenic variation is to evade the adaptive immune response and to a lesser extent, the innate immune response [47]. Our data demonstrated that MBA size variants were generated in all animals, not just those in which anti-ureaplasma IgG antibodies were detected. This suggests that MBA size variation was not driven by the development of a host humoral response, nor did it prevent recognition by host pattern recognition receptors. However, continual size variation of the MBA may prevent the eradication of ureaplasmas due to changes to epitopes within the repeat region of the protein. Because IgG is unable to cross the placenta in sheep, the ureaplasma-specific antibodies detected in maternal sera were most likely generated in response to ureaplasma invasion of maternal tissues, such as the decidua. As expected, the sheep anti-ureaplasma IgG antibodies were predominantly reactive against the MBA, however a 45 kDa protein expressed by MBA-negative ureaplasma clones was also found to be immunogenic. Previous studies have demonstrated that the presence of serum antibodies against ureaplasmas was more associated with preterm birth, low birth weight, stillbirth, neonatal respiratory disease and fetal death when compared to patients without anti-ureaplasma antibodies [52], [53]. Quinn [52] reported that a fetal antibody response to *U. urealyticum* occurred in 77.3% of stillbirths, 58.3% of respiratory disease cases, 69.3% of neonatal deaths, 80.4% of term neonates with complications, but only in 6.5% of healthy, term neonates (p≤0.001). Additionally, Horowitz *et al.* [53] reported that the rates of preterm birth and fetal death were significantly higher in women with antibodies against *U. urealyticum* compared to those without these antibodies (90% vs. 43% (p = 0.006); and 85% vs. 28% (p = 0.001) respectively). In our experiment we did not observe any differences in fetal outcomes in animals with/with-out anti-ureaplasma IgG, except for the presence of meconium-stained AF, which was increased in those animals with IgG antibodies (data not presented). Interestingly, only two fetuses developed IgG antibodies in response to chronic ureaplasma infection.

It is well documented that intra-uterine infection/inflammation is associated with adverse pregnancy outcomes (especially preterm birth [54]), via mechanisms reviewed elsewhere [7], [55], [56]. Specifically, elevation of pro-inflammatory cytokines/chemokines such as IL-1β, Il-6, IL-8 and TNF-α within the AF and fetal membranes have been associated with preterm delivery and chorioamnionitis [57]–[62]. In this study, intra-amniotic infection with either the avirulent-derived or virulent-derived ureaplasma clone tended to result in the increased expression of IL-1β, IL-6 and IL-8 within chorioamnion tissue; however, there were high levels of intra-animal variation, suggesting variability in the host immune response. Surprisingly, we did not observe any increases in TLR1, TLR2 or TLR6 expression, which are the pattern recognition receptors through which the MBA activates nuclear factor kappaB [63]. Reyes *et al.* [64] also noted variability in the innate immune response after inoculation of *U. parvum* into the bladder of Fischer 344 rats. They demonstrated that the severity of urinary tract infection was associated with distinct urine cytokine profiles. Asymptomatic urinary tract infection was associated with elevation of interferon-γ, IL-18 and monocyte chemotactic protein-1, whereas complicated urinary tract infection with struvite formation was characterised by increased IL-1α, IL-1β and growth related oncogene/keratinocyte chemoattractant (analogous to human IL-8). Kasper *et al.* [65] also found differences in the expression of IL-8 within human AF infected with *U. parvum* and determined that bacterial load significantly influenced the levels of AF IL-8. In our study, differences in IL-1β, IL-6 and IL-8 expression did not correlate with ureaplasma CFU/mL or gram of tissue, or the severity of histological chorioamnionitis. Others have demonstrated that intra-amniotic ureaplasma infection did not result in increased levels of any tested cytokines (IL-1β, IL-1 receptor antagonist, IL-4, IL-6 and TNF-α [9]). Also, *in vitro* stimulation experiments, in which human choriodecidua or fetal membrane tissues were stimulated with ureaplasmas, demonstrated a T helper-2 dominant cytokine response (characterized by IL-10 production, [66], [67]). Taken together, these data suggest that the host innate immune response to intra-amniotic ureaplasma infection is not uniform, and this could account for the variety of outcomes associated with *in-utero* ureaplasma infection in humans.

We found that the animals with maternal anti-ureaplasma IgG antibodies had significantly higher levels of IL-1β, IL-6 and IL-8 within chorioamnion tissue, when compared to IgG negative animals. We predict that a strong pro-inflammatory innate response within the chorioamnion may have induced a humoral immune response in these animals. This is the first time a correlation between the innate and adaptive immune response during intra-amniotic ureaplasma infection has been described and is a finding unique to this animal model.

In conclusion, we have demonstrated that *U. parvum* avirulent-derived and virulent-derived clones are able to chronically colonise the AF of pregnant sheep and cause histological chorioamnionitis. Our data suggest that ureaplasmas may not be intrinsically virulent or avirulent; and it appears to be more likely that the host immune response generated against intra-amniotic ureaplasma infection is a key determinant of adverse pregnancy outcomes. Size variation of the MBA did not correlate with different histological outcomes, and MBA size variation occurred in all animals, regardless of the intensity of the innate and adaptive immune responses. This suggests that MBA size variability did not prevent recognition by host pattern recognition receptors. However, it may prevent the host immune response from eradicating ureaplasmas from the amniotic cavity and thus play a role in the virulence of these microorganisms.
